# Improved UNet Deep Learning Model for Automatic Detection of Lung Cancer Nodules

**DOI:** 10.1155/2023/9739264

**Published:** 2023-01-30

**Authors:** Vinay Kumar, Baraa Riyadh Altahan, Tariq Rasheed, Prabhdeep Singh, Devpriya Soni, Hashem O. Alsaab, Fardin Ahmadi

**Affiliations:** ^1^Department of Computer Science, Dyal Singh Evening College (University of Delhi), Delhi 110003, India; ^2^Department of Medical Instrumentation Techniques Engineering, Al-Mustaqbal University College, Hilla, Babylon, Iraq; ^3^Department of English, College of Science and Humanities, Al-Kharj Prince Sattam Bin Abdulaziz University, Al-Kharj 11942, Saudi Arabia; ^4^Department of Computer Science & Engineering, Graphic Era Deemed to be University, Dehradun 248002, Uttarakhand, India; ^5^Associate professor Department of Computer Science & Engineering and Information Technology, Jaypee Institute of Information Technology, Noida, India; ^6^Department of Pharmaceutics and Pharmaceutical Technology, Taif University, P.O. Box 11099, Taif 21944, Saudi Arabia; ^7^Addiction and Neuroscience Research Unit, Taif University, Taif 21944, Saudi Arabia; ^8^Computer Science Faculty, University Rana University, Kabul, Afghanistan

## Abstract

Uncontrolled cell growth in the two spongy lung organs in the chest is the most prevalent kind of cancer. When cells from the lungs spread to other tissues and organs, this is referred to as metastasis. This work uses image processing, deep learning, and metaheuristics to identify cancer in its early stages. At this point, a new convolutional neural network is constructed. The predator technique has the potential to increase network architecture and accuracy. Deep learning identified lung cancer spinal metastases in as energy consumption increased CT readings for lung cancer bone metastases decreased. Qualified physicians, on the other hand, discovered 71.14 and 74.60 percent of targets with energies of 140 and 60 keV, respectively, whereas the proposed model gives 76.51 and 81.58 percent, respectively. Expert physicians' detection rate was 74.60 percent lower than deep learning's detection rate of 81.58 percent. The proposed method has the highest accuracy, sensitivity, and specificity (93.4, 98.4, and 97.1 percent, respectively), as well as the lowest error rate (1.6 percent). Finally, in lung segmentation, the proposed model outperforms the CNN model. High-intensity energy-spectral CT images are more difficult to segment than low-intensity energy-spectral CT images.

## 1. Introduction

Lung cancer is defined by uncontrolled cell proliferation. Tumours form when aberrant cells proliferate in areas they should not. Lung disorders are growing more widespread in contemporary, industrialised cities, necessitating improved early detection procedures. Pulmonary carcinoma is one of the most serious types of lung cancer. Cancer causes one-third of all deaths. Approximately 80% of people with this cancer may live a normal life for the first five years after diagnosis. Pollution is a major contributor to this illness. Lung disease must be discovered and treated as soon as possible to enhance the chances of a cure. Lung cancer is often detected via radiography and CT scans, as well as a biopsy, bronchoscopy, and breast mucosa examination. A pulmonary nodule is an opaque, spherical lesion that develops inside the lung tissue. Small spherical radiographic opacities in the liver or lungs are known as nodules. Lung diseases are currently being studied in a variety of ways, with more to come. The substantial removal of lung tissue, the vast number of radiographic images, and the complex and uneven structure may make an accurate diagnosis difficult. CADAS is a computer-based system that assists clinicians in diagnosing medical problems [1, 2]. These tools provide images of potentially dangerous situations to help radiologists make the best diagnosis. It is best to use a machine to increase sensitivity and decrease positive mistakes. Several works of art have been shown here.

## 2. Related Work

A lung cancer diagnostic tool was built using MSE, MFE, RCMFE, and MPE. As an algorithmic approach, multiscale fuzzy entropy was applied. The standard deviation of the most accurate MFE-based texture properties was 1.95E 50. The simulation results revealed that RCMFE measures excelled their rivals when it came to researching lung cancer dynamics. They developed an algorithm for detecting lung cancer in CT scans. They analysed lung CT data using LDA and a deep neural network. An LDR was used to reduce the size of the CT lung imaging features [3]. When the data was collected, it was classified as benign or malignant. It was enhanced by using a modified gravitational search approach on CT images to boost accuracy (MGSA). It leverages images to construct a system for quickly recognising lung cancer with the least amount of human touch. This technique retained discriminative blocks while effectively illuminating deep features [4]. A global WSI description was generated after collecting characteristics and choosing context-aware blocks. Then, it was categorised using a random forest classifier. The outcomes of the investigation proved the method's efficacy. It was a unique method for detecting lung cancer. Its purpose was to decrease misclassifications. After decreasing noise with weighted mean histogram equalization, enhanced profuse clustering was used to increase image quality (IPCT). Deep learning predicted lung cancer by collecting spectral data from the study region [5]. The simulation results indicate that the suggested strategy is effective and efficient, but with certain limitations. There are several methods for detecting lung cancer in the literature. Each has advantages and disadvantages. This study demonstrates how deep learning and metaheuristics might improve lung cancer detection systems.

**Figure 1:**
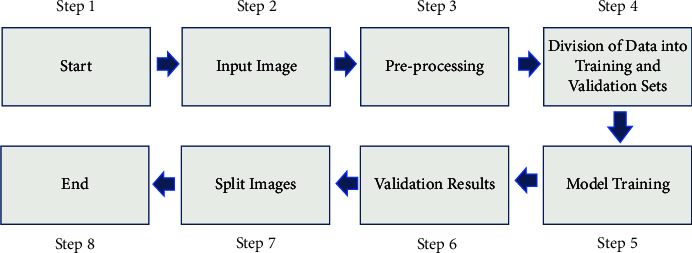
The detection of image by the improved UNet model.

Therefore, since the circulatory system is involved in the process of bone metastatic spread, lung cancer may spread to the bones. This is a symptom of advanced cancer [6–8]. The osteolytic disease affects 10–15% of lung cancer patients. Spinal cancer may spread in a variety of ways. Back discomfort and neurological impairment are caused when CSF tumour cells enter the thoracic spine from the back or neck-thoracic junction [9]. After spiral and multislice CT, energy/spectral CT is a multiparameter imaging technology. It has a multiparameter imaging capability. It is used in vascular imaging to reduce metal artefacts and expose fine structures [10, 11]. Deep learning employs artificial neural networks (ANNs) to train computers to think and learn in the same way that people do [12]. Recognition of text automatically because of memory, parameter sharing, and Turing completeness, recurrent neural networks may learn nonlinear sequences. Frame-by-frame, the extraction of RNN and CNN saves time and money [13]. Most image segmentation algorithms use two layers: receptive field constriction and feature map expansion. Computed tomography (CT), particularly dual-energy spectral computed tomography, has grown in popularity in recent years. It is an excellent resource for collecting knowledge that is both generic and. A dual-energy CT scan was first proposed in 1973, but it did not become widely used for several decades due to methodological and technological obstacles. The creation of the first dual-source CT system occurred in 2006. This device, which employs two unique X-ray energy spectra, may be useful in distinguishing between different types of materials [14–16]. An energy-spectral CT scan may be used to detect lung cancer spinal metastases. As a result, SNR and contrast were used to verify its accuracy. The detection rate was used to compare the results of clinicians with the suggested model. The study's goal was to develop a clinical standard for lung cancer bone metastases.  Figure 1 shows the segmentation approach used by the Improved UNet model [17].

The model may be narrowed or broadened. Both channels are symmetrical and gather and analyse data. Data characteristics are derived using constraints and expansions. In the contraction approach, which comes after 2 × 2 pooling, the expansion route is upsampled, while the contraction path is mirror mapped. The model combines upsampled and mirror-mapped image data. This enhanced visual quality, however, cuts the feature channel in half. Feature vector submission to the network output layer (or output feature map). The convolution block employs data characteristics and an activation function with a 256-pixel input resolution. ReLu employs a hyperparameter dilation interval as the function of activation to estimate dilation size. The contraction approach makes use of a maximum pooling of two. The number of feature channels is doubled when the image is downscaled. Three convolutional blocks and a one-to-one convolutional layer were used. A triple convolution structure is used to quadruple image resolution while halving feature channels. A mirror map joins the high and low information levels. A data layer with several channels that encourages nonlinearity. Here, are some of the study's key findings: images from lung CT scans may be used to diagnose lung cancer. For cancer diagnosis, convolutional neural networks need a certain structure. The marine predator's approach is a unique metaheuristic that was used to improve how well the convolutional neural network worked.

## 3. Proposed Model

**Figure 2:**
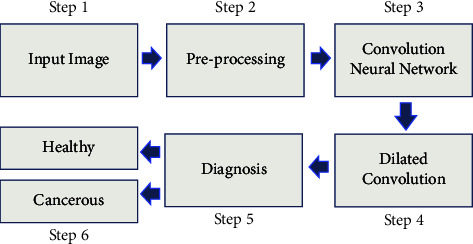
The graphical overview of the recommended technique.

CNNs are likely to become one of the most frequently utilised medical imaging technologies. CNNs do most deep learning computations in cancer screening. These deep learning algorithms take an image as input and assign relevance (learnable weights and biases) to each object/aspect inside the image, enabling them to be identified. CNN processing is, therefore, quicker than other categorised techniques. With enough practice, the CNN can recognise and recall these human-created filters and specifications. The arrangement of the brain's “visual cortex” during network development influenced human neural network connection patterns. The “receptive field” of the visual field is the area where each neuron responds to stimuli. These fields are arranged in rows and columns to fill the visual field. In this research, convolutional neural networks were used to detect lung cancer. The preferred strategy is shown in Figure 2.

In a nutshell, it safeguards CNN's brand; the preprocessed images are sent into a CNN that has been trained using the image data. Various lights and noises must be deleted before processing the lung images. Difficulties anticipating the accuracy of the final classifier a low-pass filter reduce the effect of high-frequency pixels. It is difficult to reduce noise in medical imaging. It is crucial that the image borders stay intact during noise reduction to obtain optimal image clarity. A low-pass filter is a median filter. The average brightness of the surrounding pixels is used to calculate the brightness of each output pixel [17–19]. The value of a pixel is computed by averaging pixels in the target region. Use the centre filter, which is less sensitive to toss values, to get rid of them. Light fluctuation is decreased while edge form and location are preserved [20–23]. This filter swaps the centre pixels with those surrounding them to arrange values ascendingly (*m n*). Before using the median filter, go through the image pixel by pixel and replace each value with the median value of the pixels right adjacent to each other. This must be completed before the filter is applied. The “window” of the image is a pattern of close-together pixels that gradually progresses across the image [19]. A filter was applied to the images utilised in the research.

**Figure 3:**
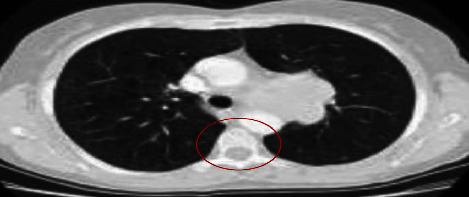
Example of noise reduction on lung images using median filtering.

**Figure 4:**
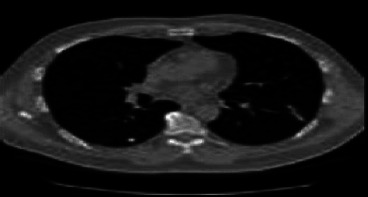
CT image that has been preprocessed.

CNN, which stands for convolutional neural networks, processes a large number of similar-sized images of the research facility [24–27]. Therefore, before being shared with CNN, all images were reduced to 227 by 227 pixels. Figures 3 and 4 show noise reduction on lung images using median filtering and a preprocessed CT image.

Properly trained networks have a lower error function. The purpose is to optimise the network's-free parameters [28–31]. The study made use of supervised training. Under this design, a manager controls and leads the network. It has a limited number of inputs and outputs [21]. The magnitude of the mistakes and the network output are compared. These are then picked in order to reduce this value. This can be done sequentially or in batches. Most people train in a row. It utilises less RAM but is less reliable since it focuses on various network aspects. The second way is more reliable, but it takes more RAM to maintain the settings. As a result, we finished the remaining jobs in batch mode. To train the database images, we used a 32-batch training approach. Before exploring for more resources, make the most of the ones you already have. This does not imply that our programme will use this information while it is running, but rather that our software will use this knowledge to learn [32–35] followed by the data collection from the previous phase, with an emphasis on detecting patterns. At this stage, a few theories may be tested, so come up with some. The basic blocks of AI are three convolutional layers and three pooling levels in a deep neural network.

**Figure 5:**
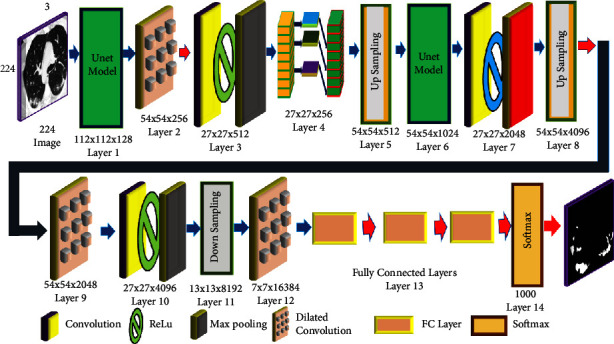
The CNN model proposed for the current situation.

The nucleus of this layer is a 3D mass of neurons in the middle. Convolutional algorithms are used to process neural inputs, reduce the depth three convolutional layers are proposed, with filter widths of 64, 32, and 128. A pooling layer was inserted after the convolutional layer to minimise the depth. This decreases the number of parameters while improving network performance. This reduces the number of output layers. It is a two-way filter. The given image is subsampled to save memory and network traffic, the smaller the input image, the lesser the sensitivity. The pooling layer, like the convolutional layer, links the outputs of many neurons [35–38], using a pooling layer when sampling may result in a smaller dataset while increasing processing performance. The image is gathered in a 2 × 2 window in this experiment.  Figure 5 displays the suggested CNN model, which involves shifting one of the window's four pixels up a layer from its previous placement.

**Figure 6:**
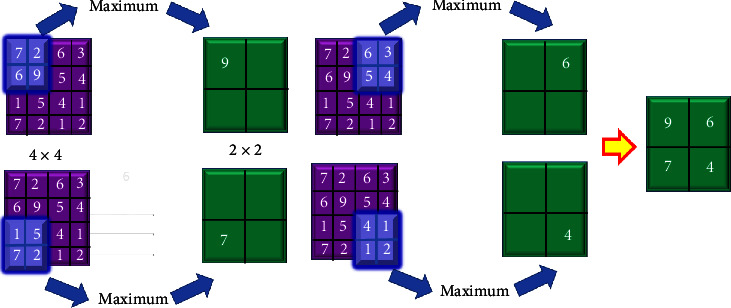
Maximum pooling operation.

**Figure 7:**
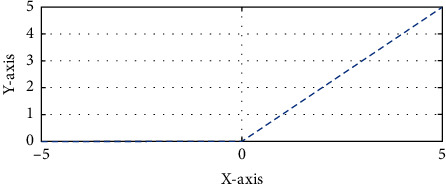
The graph below depicts the working of ReLu.

Nonlinear operations should be included after each convolutional layer. ReLu layers speed up training while maintaining accuracy. Figures 6 and 7 show the max pooling and ReLu operations, respectively, each patch of each feature map has been assigned the greatest possible value, also known as the maximum value. This number was discovered by using a pooling method called maximal pooling, sometimes known as just max pooling [25, 26]. Feature maps, which may be constructed with downsampled or pooled samples, are used to highlight the most distinguishing characteristics of a location. In contrast to the pooling technique, which emphasises the feature's general occurrence, this strategy emphasises the feature's uniqueness. The ReLu layer oversees decreasing negative activations. This layer accentuates nonlinear properties while leaving convolutional layers alone.

**Figure 8:**
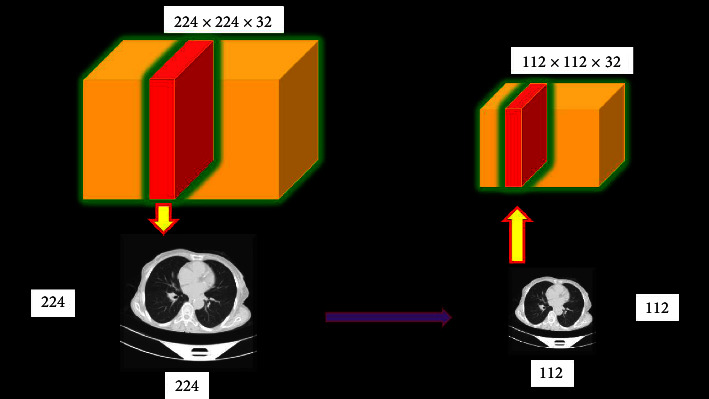
The method used to implement the dropout layer.

During training, the “dropout” layer may cause certain neurons to be eliminated from the network. The outputs of certain neurons become zero. This permits access to a different network and only employs powerful capabilities. Overfitting is avoided using the dropout approach [23]. In completely connected deep networks, convergence is more probable. An unconnected layer was employed to decrease parameter values. The dropout layer approach is shown in Figure 8.

## 4. Results

**Figure 9:**
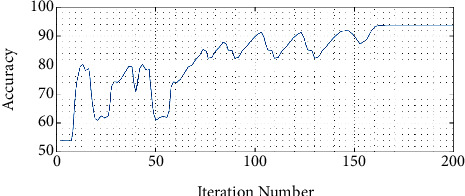
The diagnosis accuracy curve for lung cancer.

These layers provide big data sets with small axes. With enough practice, the network will be able to classify all images. The system searches for the best unknown parameters as part of the training process. Flatten, convolutional, and RMSprop layers are used in weight optimization. The activation function of an optimization function is assessed. It is possible to compare the RMSprop optimizer to a technique known as gradient descent with momentum. Both methods are used to determine the best option. The RMSprop optimizer is responsible for determining the maximum extent to which the oscillations can move in either direction. As a result of this capacity to speed up the learning process, our algorithm can now make larger horizontal jumps and settle on solutions more quickly. Nontraining images are used to evaluate the network's performance. The layer output is used to build the image feature vector. The feature vector and matrix are then compared to each data point. That's it. Probabilities must be assessed prior to categorization. Softmax, a common function, may be used to normalise probabilities (0 to 1). The optimizer RMSprop was used to optimise each variable. Deep learning algorithms in medical research uncover essential characteristics in a difficult dataset. The suggested approach uses 80 percent of the images in the dataset for training and 20 percent for testing, with no connection. Using 32-batch data, the deep neural network is trained over 200 times. The suggested approach extracts high-level characteristics in addition to employing sequential training.  Table 1 compares the recommended technique to the other choices considered. The diagnosis accuracy curve for cancer (Figure 9).

**Figure 10:**
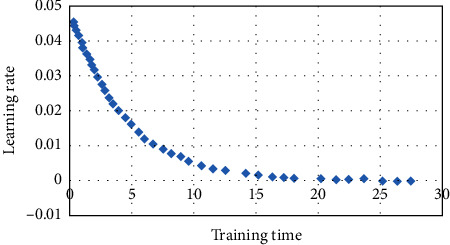
The learning rate and training time.

Deep learning is rapidly being used for image classification, object recognition, and segmentation. Deep neural networks maintained in databases may also be used to recognise images, increasing accuracy. Deep learning and machine vision have been widely researched for cancer diagnosis. Science has made major advances in this area. Lung cancer was discovered using a convolutional neural network. The results of these networks are compared. First, traditional optimizer RMSprop and metaheuristic-based techniques were used. They worked together to create the final product. The suggested MPA method was the most accurate (93.4 percent). It was preprocessed before being reduced to 126 × 126 pixels in size. The study comprised 36 lung cancer patients who had five energy-spectral CT scans. The 180 images were split into two categories: training and validation (45 images). There were three types of data used: training, testing, and validation. It was constructed using derivatives. The final image was compared to the original. Every business requires data collection and image processing. To determine which focus had the largest layer, the biggest-layer entire tumour area approach was used. Focusing on the centre of the lesion this was surrounded by bone fragments, calcification, and necrosis, reduced damage. Each ROI's CT value was utilised to generate the focus' energy spectral curve.  Figure 10 shows how training sessions have been shortened. Little new knowledge was retained after just 24 hours. After 20 repetitions, this rate dropped to zero.

In both validation and training sets, it outperforms other networks in terms of loss function and dice coefficient, showing that it is more effective. The invalidation set loss function is greater than the training set loss function. The dice coefficients in both groups were comparable (See Table 2).

For our research, we employed Improved UNet threshold-based, boundary-based, and theory-based approaches are often used for lung CT segmentation [24, 25]. A black area on a lung CT scan indicates that they are inflated. In CT images, the target area is difficult to distinguish from the surrounding lung parenchyma, and blood vessels and tiny cavities are never considered. The energy-spectral CT image was segmented using the DC-U-Net model. Figures 6 and 7 provide before and after images of the occurrence. Even though the lung was not apparent in the DC-U-Net images, blood vessels impacted the segmentation border. Increasing the amount of the training dataset minimizes errors but lengthens training time. When it comes to lung cancer bone metastases different amounts of energy yield different CT findings. Figure 8 at 90–140 keV shows that lung cancer bone metastases increased while CT value and slope decreased.  Table 3 shows that the focus detection rate was higher at 60 keV than at 140 kVp. The rate of detection by a clinically trained doctor and a deep learning system was not very different.

Early detection of lung cancer bone metastases is difficult; the pain usually implies a more severe illness. Lung cancer patients often have bone metastases, pathological fractures, and hypercalcemia. A three-dimensional CNN-based approach may increase lung nodule identification accuracy [26]. The test was successful. In clinical diagnostics, isotope scans are often used to locate bone metastases. Low specificity but high sensitivity Bone tumours may be detected by energy-spectral CT [27, 28]. It takes advantage of differences in X-ray absorption by various substances at various energy levels to deliver additional information and enhance image quality. Another important feature is the ability to analyse tiny foci subjectively and quantitatively while minimising ray hardening artifacts. K-edge imaging, with its multienergy spectrum properties, minimizes radiation and contrast agent use while boosting soft-tissue contrast. Soft and hard tissues with the same light absorption coefficient have become more contrasted in low-energy areas. Intervals are used by DC to widen the system's vision. DC-U-Net increases information extraction without adjusting image parameters [15, 16].

We wanted to see how quickly deep learning could detect lung cancer spinal metastases. To generate the final DC-U-Net models, energy-spectral CT images of lung cancer patients were used. Then, we looked at several CT images. The DC-U-Net model outperformed CNN in identifying lung shape. It may therefore be possible to distinguish between the lung and other organs using an energy-spectral CT image [17, 19]. Extending the CT scan and using the rank-sum test may help identify lung cancer and multiple myeloma bone metastases. A low-dose computed tomography scan (LDCT) is the only currently approved screening test for lung cancer (also called a low-dose CT scan or LDCT). An X-ray scanner performs a low-dose computed tomography (LDCT) scan to obtain complete images of the lungs while exposing the patient to the absolute lowest amount of radiation. Regardless, it should just take a few minutes and there should be no discomfort throughout. The higher the energy at 60 keV, the higher the SNR and CNR, these rates were almost identical at 140 kVp and 40 keV, suggesting that the deep learning system could accurately detect focus [20–23].

## 5. Conclusions

The cancer incidence has grown due to a century of poor living circumstances and harmful behaviours. As a result, scientists are striving to develop a cure. Early discovery makes this disorder less deadly and easier to cure. Using this approach, lung cancer may be discovered early. This research demonstrates a self-learning deep neural network approach for CT lung imaging based on reinforcement learning. When deep networks retrieve high-level characteristics, classification and diagnostic accuracy improve. With less storage capacity, speed and accuracy improved. Accuracy increases with reduced feature vector sizes. We will continue to explore ways to improve the system's performance for real-time apps. A more accurate DC-U-Net model with a lower dice coefficient removed more lungs from CT images, When the tumour was at its most advanced stage, the dice coefficient was as low as 0.440. This dataset provides a substantial quantity of data. Regardless of how it was discovered here, just a small fraction of the tumour had been investigated. The volume of the tumour is roughly six times smaller on a pixel scale than it would be in millilitres. The low dice coefficient is very certainly due to the undersegmentation of a microscopic kidney tumour. This is a very real possibility. This owes, in part, to the fact that smaller kidney tumours are more difficult to detect in their early stages of development rather than in the first phases of development. The training sessions also influenced the learning rate of this model. The positive CT value of the lung cancer bone metastatic focus demonstrated this. SNR and CNR both peaked at less than 60 keV. In terms of performance, the deep learning system was comparable to that of a doctor. This study, on the other hand, contains significant shortcomings. The study makes use of a tiny sample size and a crude scan. More studies on energy/spectral CT for lung cancer bone metastases may be necessary. Energy-based and spectral CT scans are recommended by researchers for detecting lung cancer bone metastases.

## Figures and Tables

**Table 1:** Comparison of performance of the proposed model with other models.

Methods	Sensitivity	Error	Accuracy	Specificity
Proposed method	98.4	1.6	95.5	97.1
ResNet101	92.9	12.0	89.1	88.3
ResNet-18	93.4	6.6	89.3	68.5
GoogLeNet	71.5	31.8	68.2	48.2
AlexNet	92.7	11.4	88.6	72.7
VGG-19	91.6	17.3	82.7	73.6

**Table 2:** The results of the experiments are compared to each other.

	Data	Convolution neural network	Improved UNet
Loss function	Train	63.8	57.5
	Validate	81.5	81.2

Dice metrics	Train	96.2	97.3
	Validate	95.4	96.2

**Table 3:** Comparison of the cancer detection rate achieved by the proposed model and doctors' report.

The energy level	Doctors report			The proposed model result		
	Cases identified	Total	Success rate (%)	Cases identified	Total	Success rate (%)
140	212	298	71.14	228	298	76.51
60	235	315	74.60	257	315	81.58

## Data Availability

A collection of lung images is taken from the CT scan and it will be provided whenever required.
